# Bacterial Coinfection and Superinfection in Respiratory Syncytial Virus-Associated Acute Respiratory Illness: Prevalence, Pathogens, Initial Antibiotic-Prescribing Patterns and Outcomes

**DOI:** 10.3390/tropicalmed8030148

**Published:** 2023-02-27

**Authors:** Phunsup Wongsurakiat, Siwadol Sunhapanit, Nisa Muangman

**Affiliations:** 1 Division of Respiratory Diseases and Tuberculosis, Department of Medicine, Faculty of Medicine Siriraj Hospital, Mahidol University, 2 Wanglang Road, Bangkoknoi, Bangkok 10700, Thailand; 2 Division of Pulmonary Medicine and Pulmonary Critical Care, Faculty of Medicine, Vajira Hospital, Navamindradhiraj University, Bangkok 10300, Thailand; 3 Diagnostic Division, Department of Radiology, Faculty of Medicine Siriraj Hospital, Mahidol University, 2 Wanglang Road, Bangkoknoi, Bangkok 10700, Thailand

**Keywords:** respiratory syncytial virus, acute respiratory illness, adult non-immunocompromised patients, bacterial coinfection, bacterial superinfection, hospital-free days, mortality, outcomes, pneumonia

## Abstract

We aimed to determine the prevalence of bacterial coinfection (CoBact) and bacterial superinfection (SuperBact), the causative pathogens, the initial antibiotic-prescribing practice, and the associated clinical outcomes of hospitalized patients with respiratory syncytial virus-associated acute respiratory illness (RSV-ARI). This retrospective study included 175 adults with RSV-ARI, virologically confirmed via RT-PCR, during the period 2014–2019. Thirty (17.1%) patients had CoBact, and 18 (10.3%) had SuperBact. The independent factors associated with CoBact were invasive mechanical ventilation (OR: 12.1, 95% CI: 4.7–31.4; *p* < 0.001) and neutrophilia (OR: 3.3, 95% CI: 1.3–8.5; *p* = 0.01). The independent factors associated with SuperBact were invasive mechanical ventilation (aHR: 7.2, 95% CI: 2.4–21.1; *p* < 0.001) and systemic corticosteroids (aHR: 3.1, 95% CI: 1.2–8.1; *p* = 0.02). CoBact was associated with higher mortality compared to patients without CoBact (16.7% vs. 5.5%, *p* = 0.05). Similarly, SuperBact was associated with higher mortality compared to patients without SuperBact (38.9% vs. 3.8%, *p* < 0.001). The most common CoBact pathogen identified was *Pseudomonas aeruginosa* (30%), followed by *Staphylococcus aureus* (23.3%). The most common SuperBact pathogen identified was *Acinetobacter* spp. (44.4%), followed by ESBL-positive Enterobacteriaceae (33.3%). Twenty-two (100%) pathogens were potentially drug-resistant bacteria. In patients without CoBact, there was no difference in mortality between patients who received an initial antibiotic treatment of <5 days or ≥5 days.

## 1. Introduction

Respiratory syncytial virus-associated acute respiratory illness (RSV-ARI) is common in adults, particularly older adults, in chronic cardiopulmonary conditions, and in adults that are immunocompromised [1,2,3]. Approximately 14.5% of RSV-ARI cases required hospital admission [4,5].

We recently reported that bacterial infection was an independent factor associated with mortality in adult non-immunocompromised patients hospitalized with RSV-ARI [6]. The interaction between virus and bacterial infection has been widely recognized in patients with viral respiratory infection. In hospitalized patients with influenza and COVID-19, a large number of clinical studies have reported on bacterial infection and its impact on outcomes [7,8,9]. Bacterial coinfection is well described with influenza, with the frequency of coinfection ranging from 2% to 65%. The most frequently identified bacteria are *Staphylococcus aureus*, *Streptococcus pneumoniae*, *Haemophilus influenzae,* and *Streptococcus pyogenes*. The frequency of bacterial superinfection in influenza ranges from 0.5% to 38%. The most frequently detected pathogens are *Acinetobacter baumannii*, *Staphylococcus aureus*, *Klebsiella pneumoniae*, and *Pseudomonas. aeruginosa.*

The prevalence of bacterial coinfection in COVID-19 ranges from 5% to 11%. The most frequently identified bacteria are *Klebsiella pneumoniae*, *Streptococcus pneumoniae*, and *Staphylococcus aureus.* The frequency of bacterial superinfection ranges from 13% to 28%. The most frequently identified bacteria are *Acinetobacter* spp., *Pseudomonas*, and *Escherichia coli.*

The common risk factors reported for coexisting bacterial infection in respiratory viral illness were older age, comorbidities, severity of illness (invasive mechanical ventilation, acute respiratory distress syndrome), and impaired immune response including immunosuppressive treatments.

However, although the disease burden is comparable [3,10,11,12], there is scarce data on the frequency of bacterial coinfection, superinfection, and associated clinical outcomes among patients with RSV-ARI [10,13,14,15,16]. Furthermore, the American Thoracic Society/Infectious Diseases Society of America (ATS/IDSA) guideline for community-acquired pneumonia (CAP) recommends that standard antibacterial therapy be initially prescribed to patients with CAP who test positive for influenza and could consider earlier discontinuation of antibiotics if there is no evidence of bacterial infection and the patient is clinically stable [17]. However, the impact of initial antibiotic-prescribing practice in RSV-ARI is still unclear. 

This study set forth to determine the prevalence of bacterial coinfection and bacterial superinfection, the causative pathogens, the initial antibiotic-prescribing practice and the associated clinical outcomes of patients hospitalized with RSV-ARI.

## 2. Materials and Methods 

This study is a secondary analysis of data collected from a retrospective study of a cohort of adults hospitalized with RSV-ARI from January 2014 to April 2019 at a university-based hospital in Bangkok, Thailand, the design of which was previously described [6]. Briefly, patients with RSV infection were identified from the inpatient database of our hospital using the ICD-10 code related to RSV infection. RSV infection was defined by a positive reverse transcription polymerase chain reaction (RT-PCR) on any of the respiratory samples. Patients with RSV infection together with all of the following criteria were included: aged 18 years or more; admitted from outside the hospital with ARI (≥2 respiratory symptoms, including pleuritic chest pain, dyspnea, cough, and/or respiratory distress); and diagnosis of RSV infection within 48 h after admission. Patients who had received immunosuppressants or long-term corticosteroid therapy, who had concomitant acquired immunodeficiency syndrome, or who were pregnant were excluded. 

### 2.1. Data Collection

The medical records of all patients were reviewed. In all cases, a protocol for data collection was applied. The data collected included age, sex, functional status, comorbidities, and time of hospital admission, presenting signs and symptoms, vital signs, mental status, the need for mechanical ventilation or vasopressors within 48 h after hospital admission, chest radiographic findings, laboratory data and arterial blood gas analysis. PaO_2_ was inferred from SpO_2_ if the arterial blood gas analysis was not available [18].

All microbiological studies for bacterial infection during the course of hospitalization were recorded, including sputum cultures, blood cultures, BAL cultures, or pleural fluid cultures. The etiologic diagnosis was considered positive in the following situations: a predominant microorganism isolated from a sputum sample with moderate or high quantity, isolation of a respiratory pathogen in a usually sterile specimen, or bacterial growth in BAL fluid (≥10^4^ cfu/mL).

### 2.2. Medication Treatment 

The following medications prescribed during hospitalization were recorded:

Initial antibiotic treatment prescribed within the first 24 h after admission. 

Ribavirin treatment prescribed with ≥1 doses during admission.

Systemic corticosteroids used ≥24 h after admission.

Inhaled bronchodilator. 

### 2.3. Definitions 

**Severe ARI** was defined using ATS/IDSA criteria for severe CAP [17] (Table S1). 

**Bacterial coinfection**: ≥1 positive cultures from blood and/or a respiratory sample collected within 48 h after hospitalization. 

**Bacterial superinfection**: ≥1 positive cultures from blood and/or a respiratory sample collected >48 h after hospitalization. 

**Adequate initial antibiotic therapy**: Initial antibiotic treatment with at least one agent to which all recovered isolates were susceptible in vitro.

**Guideline-concordant antibiotic therapy:** Initial antibiotic treatment prescribed were concordant with ATS/IDSA guideline for CAP as outlined in Table S2. 

### 2.4. Outcomes 

The primary outcome was all-cause mortality within 30 days after admission. The secondary outcome was the duration of hospitalization by assessing the number of days alive and outside the hospital (hospital-free days) within 30 days after hospital admission. 

### 2.5. Statistical Analysis

Descriptive analysis was performed. Discrete variables are expressed as number and percentage (%), and continuous variables as either mean ± standard deviation (SD) or median and interquartile range (IQR). Proportions were compared using a chi-square test or Fisher’s exact test for categorical variables, and a nonparametric Mann–Whitney U-test or unpaired *t*-test for continuous variables. The Kolmogorov–Smirnov test was used to evaluate data distribution.

Multivariate analysis by logistic regression analysis was performed to determine the independent factors associated with bacterial coinfection. The dependent variable was the rate of bacterial coinfection, and the independent variables were variables that were associated with bacterial coinfection, including in the univariate analysis (*p* < 0.1) and biologically relevant confounders (age). 

Factors affecting bacterial superinfection were analyzed using time-to-event analyses. Time to bacterial superinfection was defined as the time from hospital admission to the date of occurrence of the first bacterial superinfection within 30 days after admission. Univariate and multivariate Cox regression analyses were performed to determine independent factors associated with the occurrence of bacterial superinfection. Factors with a *p* value of <0.1 in the univariate analysis, age, and ribavirin treatment were entered into a multiple variable Cox regression model. A higher hazard ratio (HR > 1) indicates a higher probability of bacterial superinfection. 

All statistical analyses were two-sided, and a *p* value of <0.05 was considered statistically significant. All statistical analyses were performed using SPSS Statistics software version 20 (SPSS, Inc., Chicago, IL, USA).

## 3. Results

A total of 175 adult patients with community-acquired RSV-ARI were included in this study. The mean age was 76 ± 12.7 years. Clinical characteristics, functional status, comorbidities, chest radiographic findings, laboratory data, and prevalence of bacterial coinfection and bacterial superinfection are shown in Table 1. 

Seventy-eight (44.6%) patients met the diagnostic criteria for severe ARI. The prevalence of bacterial coinfection and bacterial superinfection according to the severity of ARI is shown in Table 2.

### 3.1. Medication Treatment

Fifty-two (29.7%) patients received systemic corticosteroids during hospital admission. The prevalence of bacterial coinfection and bacterial superinfection according to the medication treatments is shown in Table 2. 

### 3.2. Bacterial Infection

Blood cultures were performed in 135 (77.1%) patients and were positive in 3 (2.2%) patients. Thirty (17.1%) patients had bacterial coinfection, and 18 (10.3%) had bacterial superinfection, as follows: two (11.1%) had hospital-acquired pneumonia (HAP), seven (38.9%) ventilated HAP, and nine (50.0%) ventilator-associated pneumonia (VAP). Seven (4%) patients had both bacterial coinfection and bacterial superinfection. 

Invasive mechanical ventilation (odds ratio: 12.1, 95% CI: 4.7–31.4; *p* < 0.001) and absolute neutrophil count of >6.9 cells × 10^3^/mm^3^ (odds ratio: 3.3, 95% CI: 1.3–8.5; *p* = 0.01) were found to be independent factors associated with bacterial coinfection. The independent factors associated with bacterial superinfection were invasive mechanical ventilation (aHR: 7.2, 95% CI: 2.4–21.1; *p* < 0.001) and systemic corticosteroids (aHR: 3.1, 95% CI: 1.2–8.1; *p* = 0.02), as shown in Table 3. 

### 3.3. Outcomes

Overall mortality within 30 days was 7.4%. Bacterial coinfection was associated with higher mortality compared to patients without bacterial coinfection (16.7% vs. 5.5%, *p* = 0.05). Similarly, bacterial superinfection was associated with higher mortality compared to patients without bacterial superinfection (38.9% vs. 3.8%, *p* < 0.001), as shown in Table 2.

Bacterial coinfection was associated with fewer hospital-free days compared to patients without bacterial coinfection [16 (3–20.2) days vs. 21 (14.5 to 24) days, *p* = 0.002). Bacterial superinfection was associated with fewer hospital-free days compared to patients without bacterial superinfection [0 (0 to 5.2) days vs. 21 (16.5 to 24) days *p* < 0.001)], as shown in Table 2. 

### 3.4. Causative Pathogens

The most common bacterial coinfection pathogen identified was *Pseudomonas aeruginosa* (nine patients, 30%), followed by *Staphylococcus aureus* (seven patients, 23.3%). Nineteen (63.3%) pathogens identified were potentially drug-resistant bacteria, six (20%) were multidrug-resistant (MDR) bacteria, and two (6.7%) were extensively drug-resistant (XDR) bacteria. Polymicrobial coinfection was identified in five (16.7%) patients. The most common bacterial superinfection pathogen identified was *Acinetobacter* spp. (eight patients, 44.4%), followed by ESBL-positive Enterobacteriaceae (six patients, 33.3%). Twenty-three (100%) of the pathogens identified were potentially drug-resistant bacteria, eight (34.8%) were MDR bacteria, and eight (34.8%) were XDR bacteria. Polymicrobial superinfection was identified in six (33.3%) patients, as shown in Table 4.

### 3.5. Initial Antibiotic-Prescribing Patterns

Initial antibiotic treatment was prescribed in 150 (85.7%) patients. The initial antibiotic regimens are shown in Table 5. Of the 175 patients with RSV-ARI, 81 (46.3%) received initial antibiotic regimens that were concordant with the ATS/IDSA guideline [17]. The initial antibiotic regimens included antipseudomonas β-lactams in 58 (33.1%) patients, macrolides in 79 (45.1%) patients and atypical pathogen coverage in 106 (60.6%) patients. There was no association between these initial antibiotic regimens and the mortality of all patients with RSV-ARI. In patients with bacterial coinfection, mortality in patients who received inadequate initial antibiotic treatment was higher than in patients who received adequate initial antibiotic treatment (28.6% vs. 13%, *p* = 0.56). There was no association between initial antibiotic regimens and the mortality of patients without evidence of bacterial coinfection, except for an initial antibiotic regimen that included antipseudomonas β-lactams, which was associated with higher mortality.

The average duration of initial antibiotic treatment in patients with bacterial coinfection was longer than in patients without bacterial coinfection [7 (IQR: 5–7.2) days vs. 6 (IQR:3–7) days, *p =* 0.01]. However, 91 (62.8%) patients without bacterial coinfection received initial antibiotic treatment for 5 days or more. There was no association between the duration of initial antibiotic treatment and the mortality of patients without evidence of bacterial coinfection. There was no difference in mortality, along with increased hospital-free days in patients who received a shorter duration of initial antibiotic treatment (<5 days) compared to patients who received a longer duration of initial antibiotic treatment (≥5 days), as shown in Table 5 and Table S3.

There was no association between initial antibiotic treatment regimens and hospital-free days of all patients with RSV-ARI, except the initial antibiotic regimen that included antipseudomonas β-lactams, which was associated with fewer hospital-free days. There was no association between initial antibiotic regimens and hospital-free days for patients without bacterial coinfection, as shown in Table S3.

## 4. Discussion 

Bacterial coinfection was documented in 17.1% of patients hospitalized with RSV-ARI in this study. Previous reports documented bacterial coinfection in 9–30% of patients with RSV infection [10,13,14,15]. However, the bacterial testing was left to the discretion of the attending physician. Microbiological diagnosis of bacterial infection may vary depending on the severity of the patient, patient cooperation, and may be limited by sampling techniques. In addition, only culture-proven cases were reported. This may lead to underestimates of bacterial infection. 

The interaction between virus and bacterial infection has been frequently documented in patients with respiratory viral infection. The prevalence of bacterial coinfection and association with increased mortality in the present study was comparable to previous reports of bacterial infection in patients hospitalized with RSV infection [10,13,14,15] and patients with influenza infection [7]. Therefore, patients with RSV infection should be carefully assessed for possible bacterial coinfection. Our analysis revealed that invasive mechanical ventilation and neutrophilia were independently associated with bacterial coinfection. However, there were no clinical characteristics, radiology, or routine blood tests that could reliably differentiate bacterial coinfection from primary RSV-ARI. Serum procalcitonin has been reported to be used as a marker of the absence of bacterial coinfection in influenza with a high negative predictive value [19]. We did not evaluate procalcitonin in our study.

IDSA guidelines on influenza management recommend the investigation and empirical treatment of bacterial coinfection in patients with influenza who present with severe disease (respiratory failure, extensive pneumonia, fever, and hypotension), who do not improve or deteriorate after initial improvement [20]. These clinical characteristics were also identified as factors associated with bacterial coinfection in univariate and multivariate analysis in the current study. Due to the comparable prevalence of bacterial coinfection with a significant impact on mortality, along with the inability to completely exclude bacterial coinfection, this guideline should be extrapolated to patients hospitalized with RSV-ARI. According to our analysis, neutrophilia should be considered as a criterion to empirically treat bacterial coinfection. 

The most common pathogens of bacterial coinfection identified in this study were *Pseudomonas aeruginosa*, followed by *Staphylococcus aureus*. The common pathogens of bacterial infection in RSV infection in previous reports were *Streptococcus pneumoniae, Staphylococcus aureus*, *Haemophilus influenzae*, and *Pseudomonas aeruginosa* [10,14,15]. A study in Korea revealed a high prevalence of Gram-negative bacteria in patients with RSV infection [16]. Around 60% of the pathogens identified in our study were potentially drug-resistant bacteria. This pattern of causative pathogens is more similar to the pattern of causative pathogens identified in CAP patients in our hospital [21] than the common pathogens (*Streptococcus pneumoniae*, *Staphylococcus aureus*) reported in most studies of bacterial coinfection in RSV and influenza infection [10,14,15,22,23,24].

There was no association between initial antibiotic regimens and outcomes including mortality and hospital-free days in all patients with RSV-ARI. The present study documented bacterial coinfection in only 30 patients, of which there may be insufficient power to demonstrate the impact of initial antibiotic regimens on the outcomes. In patients with bacterial coinfection, the mortality of patients who received inadequate initial antibiotic treatment was twice as high as the mortality of patients who received adequate initial antibiotic treatment. The spectrum of bacterial coinfection pathogens is similar to the pattern of causative pathogens identified in patients with CAP. Therefore, the initial antibiotic treatment should be similar to the antibiotics recommended for CAP, taking into account local epidemiology and antibiotic resistance patterns. 

Due to the inability to differentiate bacterial coinfection from primary RSV-ARI and the significant risk of delaying appropriate antibiotic treatment, most patients would receive empirical antibiotic treatment. The ATS/IDSA guideline for CAP recommends that standard antibacterial therapy be initially prescribed for patients with CAP who test positive for influenza and that it should be considered to discontinue treatment at 48 to 72 h, if there is no evidence of bacterial infection and the patient is clinically stable [17]. Our study demonstrated that 60% of patients without bacterial coinfection received initial antibiotic treatment for five days or more. We used five days as the cut-off date for discontinuing initial antibiotic treatment, as it is the usual duration needed for all routine bacterial culture results. We showed that there was no difference in mortality, along with increased hospital-free days, in patients who received a shorter duration of initial antibiotic treatment (<5 days) compared to patients who received a longer duration of antibiotic treatment (≥5 days). This supports the recommendation to discontinue antibiotics if there is no evidence of a bacterial pathogen from routine bacterial cultures and early clinical stability. It is unknown whether the duration of initial antibiotic treatment could be shortened if all culture results could be obtained earlier, for example within 48–72 h.

Our study revealed that macrolides were included in initial antibiotic regimens in 45% of the patients. Macrolides exert broad-ranging anti-inflammatory and immunomodulatory properties [25], and several studies have demonstrated the beneficial effects of macrolides in viral infections [26,27]. Tahan F et al. reported that clarithromycin treatment was associated with significant reductions in the need for bronchodilator treatment, the duration of supplemental oxygen requirement, and the length of hospital stay in respiratory syncytial virus bronchiolitis [28]. However, our analysis did not support the beneficial effect of macrolides on mortality and hospital-free days in patients with primary RSV-ARI. 

Our analysis distinguished between bacterial coinfection and bacterial superinfection because of the different implications in patient management. Ten percent of the patients developed respiratory bacterial superinfection, including HAP, ventilated HAP and VAP, which are severe nosocomial infections associated with high mortality (38.9%). All identified causative pathogens were potentially drug-resistant bacteria, of which almost half were XDR bacteria. Therefore, measures to prevent hospital-acquired infections should be implemented in these patients [29]. The independent factors associated with bacterial superinfection were invasive mechanical ventilation and systemic corticosteroids. Systemic corticosteroids are frequently used to decrease bronchial obstruction and inflammation in patients with RSV infection [3,30,31]. One third of the patients in this study received systemic corticosteroids. Systemic corticosteroids may diminish humoral immunity [31]. Some studies demonstrated that adjunct corticosteroid use increased the risk of disease progression [32], secondary infections, and longer hospitalization in patients with RSV infections [10]. All things considered, systemic corticosteroids should be avoided in patients with RSV infection. An alternative treatment such as high-dose inhaled corticosteroids should be considered in patients with established indications, such as exacerbations of COPD [33].

The strengths of the present study include only non-immunocompromised patients with community-acquired ARI, with RSV infection confirmation via RT-PCR. Furthermore, multivariate analysis was used to adjust for important confounders, without missing outcome-related data. The present study also has several potential limitations. First, due to the nature of a retrospective single-center study, it is vulnerable to biases and incomplete data. Second, despite many possible confounding factors being taken into account by adjusting for them, residual confounding factors cannot be completely excluded. Third, the time from admission to the first administration of antibiotics was not evaluated in this study. It was reported to be a key predictor of outcomes. Fourth, atypical pathogens were not investigated in the present study. Fifth, the treatments after the initial antibiotic therapy were not investigated. These treatments may confound the outcome analysis. Sixth, most patients in the present study were older adults with many comorbidities; therefore, unmeasured patient preference for life-sustaining treatment might confound the outcomes.

## 5. Conclusions

Bacterial coinfection and bacterial superinfection are significant complications associated with worse outcomes and clinicians should consider possible bacterial coinfection in patients hospitalized with RSV-ARI, especially in patients with severe ARI, patients receiving invasive mechanical ventilation, or in patients with neutrophilia. There should be a strong consideration for an initial empiric antibiotic treatment. The choice of initial antibiotic treatment should be based on the local epidemiology and target pathogens responsible for CAP, taking into account local antibiotic resistance patterns. Therapy should be discontinued in less than five days if all microbiological results are negative. Systemic corticosteroids are an independent factor and should be avoided to minimize bacterial superinfection, which is often caused by potentially drug-resistant bacteria. Prospective studies are needed to confirm our retrospective results.

## Figures and Tables

**Table 1:** Baseline characteristics and associated bacterial coinfection and bacterial superinfection of all patients hospitalized with respiratory syncytial virus-associated acute respiratory illness.

	All Patients	Bacterial Coinfection			Bacterial Superinfection		
	n = 175	No n = 145	Yes n = 30	*p* Value	No n = 157	Yes n = 18	*p* Value
Year at admission:				0.08			0.02 *
2014 2015 2016 2017 2018	19 (10.9) 16 (9.1) 28 (16) 37 (21.1) 75 (42.9)	12 (8) 12 (8) 23 (15.9) 31 (21.4) 67 (46.2)	7 (23.3) 4 (13.3) 5 (16.7) 6 (20) 8 (26.7)		13 (8.3) 14 (8.9) 27 (17.2) 35 (22.3) 68 (43.3)	6 (33.3) 2 (11.1) 1 (5.6) 2 (11.1) 7 (38.9)	
Month at admission				0.3			0.29
May June July August September October November December	1 (0.6) 3 (1.7) 15 (8.6) 47 (26.9) 57 (32.6) 39 (22.3) 12 (6.9) 1 (0.6)	1 (0.69) 3 (2.1) 14 (9.6) 41 (28.3) 45 (31) 33 (22.8) 7 (4.8) 1 (0.69)	0 0 1 (3.3) 6 (20) 12 (40) 6 (20) 5 (16.7) 0		1 (0.64) 3 (1.9) 14 (8.9) 37 (23.6) 54 (34.4) 36 (22.9) 11 (7) 1 (0.64)	0 0 1 (5.6) 10 (55.6) 3 (16.7) 3 (16.7) 1 (5.6) 0	
Age, y	76 ± 12.6	76.5 ± 12.1	73.5 ± 14.5	0.23	75.7 ± 12.6	78.6 ± 12.3	0.35
Female	108 (61.7)	86 (86.2)	22 (73.3)	0.15	97 (61.8)	11 (61.1)	0.96
Comorbid: Cardiovascular diseases Neurological diseases Diabetes Chronic lung diseases Malignant diseases	81 (46.3) 58 (33.1) 64 (36.6) 50 (28.6) 16 (9.1)	70 (48.3) 51 (35.2) 58 (40) 40 (27.6) 14 (9.6)	11 (36.7) 7 (23.3) 6 (20) 10 (33.3) 2 (6.7)	0.25 0.21 0.04 * 0.53 0.74	70 (44.6) 55 (35) 59 (37.6) 41 (26.1) 16 (10.2)	11 (61.1) 3 (16.7) 5 (27.8) 9 (50) 0	0.18 0.12 0.41 0.03 * 0.23
Dependent functional status	52 (29.7)	43 (29.6)	9 (30)	0.97	49 (31.2)	3 (16.7)	0.2
eGFR ^a^ < 50 mL/min/1.73 m^2^	83 (47.4)	70 (48.3)	13 (43.3)	0.6	71 (45.2)	12 (66.7)	0.09
Chest radiograph: Infiltrates Multilobar infiltrates Pleural effusion	159 (90.9) 88 (50.3) 8 (4.6)	129 (88.9) 65 (44.8) 6 (4.1)	30 (100) 23 (76.7) 2 (6.7)	0.08 0.001 * 0.55	142 (90.4) 72 (45.9) 6 (3.8)	17 (94.4) 16 (88.9) 2 (11.1)	0.71 0.001 * 0.19
Hemoglobin < 10 g/dL	28 (16)	25 (17.2)	3 (10)	0.42	24 (15.3)	4 (22.2)	0.49
WBC count, median (IQR), cells × 10^3^/mm^3^	8.5 (6.5–11.9)	8.2 (6.2–11.7)	9.8 (8–13.4)	0.009 *	8.5 (6.4–11.9)	8.4 (6.9–13.7)	0.59
WBC count > 11 cells × 10^3^/mm^3^	54 (30.9)	43 (29.6)	11 (36.7)	0.45	49 (31.2)	5 (27.8)	0.76
Absolute neutrophil count, median (IQR), cells × 10^3^/mm^3^	6.5 (4.6–9.2)	6 (4.2–9)	7.7 (5.8–11)	0.02 *	6.2 (4.5–9.3)	6.6 (5–8.7)	0.81
Absolute neutrophil count > 6.9 cells × 10^3^/mm^3^	72 (41.1)	54 (37.2)	18 (60)	0.02 *	64 (40.8)	8 (44.4)	0.76
Absolute lymphocyte count, median (IQR), cells × 10^3^/mm^3^	1.3 (0.8–1.9)	1.3 (0.79–1.9)	1.5 (0.79–1.9)	0.34	1.3 (0.79–1.9)	1.5 (0.77–2)	0.43
Absolute lymphocyte count < 0.8 cells × 10^3^/mm^3^	44 (25.1)	36 (24.8)	8 (26.7)	0.83	39 (24.8)	5 (27.8)	1
Neutrophil-lymphocyte count ratio, median (IQR)	4.9 (3–8.4)	4.9 (2.9–8.7)	5.2 (3.1–8.7)	0.6	4.9 (3.2–8.1)	4.1 (2.7–9.9)	0.7

Data are presented as mean ± SD or n (%), unless otherwise stated. IQR = interquartile range. ^a^ Glomerular filtration rate estimated using CKD-EPI Creatinine Equation. * Statistically significant difference.

**Table 2:** Severity at admission, treatment, and associated bacterial coinfection and bacterial superinfection of all patients hospitalized with respiratory syncytial virus-associated acute respiratory illness.

	All Patients	Bacterial Coinfection			Bacterial Superinfection		
	n = 175	No n = 145	Yes n = 30	*p* Value	No n = 157	Yes n = 18	*p* Value
ICU admission	14 (8)	11 (7.6)	3 (10)	0.71	9 (5.7)	5 (27.8)	0.007 *
Invasive mechanical ventilation	36 (20.6)	18 (12.4)	18 (60)	<0.001 *	23 (14.6)	13 (72.2)	<0.001 *
Vasopressor requirement	11 (6.3)	5 (3.4)	6 (20)	0.004 *	9 (5.7)	2 (11.1)	0.61
Minor criteria ≥ 3 ^a^: Confusion/disorientation Hypotension Non-invasive ventilation PaO_2_/FiO_2_ ≤ 250 mmHg Multilobar infiltrates BUN ≥ 20 mg/dL WBC count < 4000 cells/mm^3^ Platelet count < 100,000 cells/mm^3^	56 (32) 22 (12.6) 7 (4) 21 (12) 93 (53.1) 88 (50.3) 74 (42.3) 10 (5.7) 14 (8)	44 (30.3) 15 (10.3) 3 (2) 19 (13.1) 77 (53.1) 65 (44.8) 62 (42.8) 10 (6.9) 13 (8.9)	12 (40) 7 (11.1) 4 (13.3) 2 (6.7) 16 (53.3) 23 (76.7) 12 (40) 0 1 (3.3)	0.3 0.07 0.02 * 0.38 0.98 0.001 * 0.78 0.21 0.47	49 (31.2) 18 (11.5) 6 (3.8) 20 (12.7) 84 (53.5) 72 (45.7) 64 (40.8) 9 (5.7) 13 (8.3)	7 (38.9) 4 (22.2) 1 (5.6) 1 (5.6) 9 (50) 16 (88.9) 10 (55.6) 1 (5.6) 1 (5.6)	0.51 0.25 1 0.48 0.78 0.001 * 0.23 1 1
Severe acute respiratory illness ^b^	78 (44.6)	58 (40)	20 (66.7)	<0.007 *	62 (39.5)	16 (88.9)	<0.001 *
Positive blood culture	3 (1.7)	0/110 (0)	3/25 (82.8)	0.006 *	1 (0.6)	2 (11.1)	0.04 *
Bacterial coinfection	30 (17.1)	0	30	-	23	7	0.02 *
Ribavirin treatment	99 (56.6)	79 (54.5)	20 (66.7)	0.22	83 (52.9)	16 (88.9)	0.003 *
Systemic corticosteroids use	52 (29.7)	44 (30.3)	8 (26.7)	0.69	42 (26.7)	10 (55.6)	0.01 *
Bronchodilator therapy	157 (89.7)	127 (87.6)	30 (100)	0.05 *	139 (88.5)	18 (100)	0.22
Mortality at 30 days	13 (7.4)	8 (5.5)	5 (16.7)	0.05 *	6 (3.8)	7 (38.9)	<0.001 *
Time to death	-	1.9 (0.6–5.8) ^c^		0.26	3.8 (1.2–11.9) ^c^		0.02 *
Length of stay in hospital, median (IQR), d	9 (6–15)	9 (6–13.5)	13 (8.7–21)	0.004 *	9 (6–13)	22 (15.2–37.7)	<0.001 *
Hospital-free days ^d^, median (IQR), d	20 (13–23)	21 (14.5–24)	16 (3–20.2)	0.002 *	21 (16.5–24)	0 (0–5.2)	<0.001 *

Data are presented as mean ± SD or n (%), unless otherwise stated. IQR = interquartile range. ^a^ IDSA/ATS minor criteria for severe community-acquired pneumonia [17]. ^b^ Defined by IDSA/ATS criteria for severe community-acquired pneumonia [17]. ^c^ hazard ratio (95%CI) of time to death compared bacterial coinfection with no bacterial coinfection, and bacterial superinfection with no bacterial superinfection, analyzed by Cox regression analysis. ^d^ Number of days from admission to day 30 that the patient was not admitted to the hospital. * Statistically significant difference.

**Table 3:** Univariate and multivariate analyses of potential factors associated with bacterial coinfection and bacterial superinfection.

**Factors Associated with Bacterial Coinfection**	**Univariate Model**			**Multivariate Model**		
	**Odds Ratio**	**95% CI**	***p* Value**	**Odds Ratio**	**95% CI**	***p* Value**
Age	0.98	0.95–1	0.24	-	-	-
Year of study Diabetes mellitus Confusion/disorientation	0.2 0.4 2.6	0.06–0.7 0.1–0.9 0.9–7.1	0.009 * 0.04 * 0.06	- - -	- - -	- - -
Multilobar infiltrates on chest radiograph	4	1.6–10	0.003 *	-	-	-
Vasopressor requirement	7	1.9–24.8	0.003 *	-	-	-
Invasive mechanical ventilation	10.6	4.4–25.6	<0.001 *	12.1	4.7–31.4	<0.001 *
Absolute neutrophil count > 6.9 cells × 10^3^/mm^3^	2.5	1.1–5.6	0.02 *	3.3	1.3–8.5	0.01 *
**Factor Associated with Bacterial Superinfection**	**Hazard Ratio**	**95% CI**	***p* Value**	**Hazard Ratio**	**95% CI**	***p* Value**
Age	1	0.97–1.1	0.43	-	-	-
Year of study	0.3	0.1–0.9	0.03 *	-	-	-
ICU admission	4.3	1.5–12.1	0.006 *	-	-	-
Multilobar infiltrates on chest radiograph	7.2	1.7–31.4	0.008 *	-	-	-
Ribavirin treatment	5.2	1.2–22.9	0.03 *	-	-	-
Bacterial coinfection	2.8	1.1–7.3	0.03 *	-	-	-
Systemic corticosteroids use	2.8	1.1–7.2	0.03 *	3.1	1.2–8.1	0.02 *
Invasive mechanical ventilation	9.5	3.4–26.2	<0.001 *	7.2	2.4–21.1	<0.001 *

* Statistically significant difference.

**Table 4:** Causative pathogens of bacterial coinfection and bacterial superinfection.

	Bacterial Coinfection (n = 30)			Bacterial Superinfection (n = 18)			
	Sputum	Blood	Total	HAP ^a^ (n = 2)	Ventilated HAP ^a^ (n = 7)	VAP ^b^ (n = 9)	Total
Positive blood culture	-	3	3 (10)	-	-	-	-
Etiological diagnosis:							
*Streptococcus pneumoniae*	2	1	2 (6.7)	-	-	-	-
*Staphylococcus aureus*	6	-	6 (20)	-	-	-	-
*Haemophilus influenzae*	3	-	3 (10)	-	-	-	-
Enterobacteriaceae:							
*Klebsiella pneumoniae*	3	-	3 (10	1	-	-	1 (5.6)
*Escherichia coli*	1	1	1 (3.3)	-	-	-	-
Others	-	-	-	1	-	-	1 (5.6)
*Pasteurella multocida*	1	-	1 (3.3)	-	-	-	-
Potentially drug-resistant bacteria:	18	1	19 (63.3)	3	9	11	23 (100)
*Pseudomonas aeruginosa*	9	-	9 (30)	1	1	3	5 (27.8)
ESBL-positive Enterobacteriaceae ^c^	1	-	1 (3.3)	-	4	2	6 (33.3)
*Acinetobacter* spp.	4	-	4 (13.3)	1	2	5	8 (44.4)
*Stenotrophomonas maltophilia*	3	-	3 (10)	1	-	-	1 (5.6)
Non-fermentative Gram-negative rods	-	1	1 (3.3)	-	1	1	2 (11.1)
Methicillin resistant *Staphylococcus aureus*	1	-	1 (3.3)	-	1	-	1 (5.6)
Polymicrobials	5	-	5 (16.7)	2	3	1	6 (33.3)
MDR ^d^	6	1	6 (20)	1	5	2	8 (34.8)
XDR ^e^	2	-	2 (6.7)	1	2	5	8 (34.8)

Data are presented as n (%). ^a^ Hospital-acquired pneumonia. ^b^ Ventilator-associated pneumonia (VAP). ^c^ ESBL: extended spectrum beta-lactamase. ^d^ MDR: Non-susceptible to ≥1 agent in ≥3 antimicrobial categories. ^e^ XDR: Non-susceptible to ≥1 agent in all but ≤2 antimicrobial categories.

**Table 5:** Initial antibiotic treatment and mortality within 30 days after admission of all patients hospitalized with respiratory syncytial virus-associated acute respiratory illness.

	All Patients			Bacterial Coinfection			No Bacterial Coinfection		
	Alive (n = 162)	Dead (n = 13)	*p* Value	Alive (n = 25)	Dead (n = 5)	*p* Value	Alive (n = 137)	Dead (n = 8)	*p* Value
Initial antibiotic treatment	137 (84.6)	13 (100)	0.22	25 (100)	5 (100)	-	112 (81.7)	8 (100)	0.35
Inadequate initial antibiotic treatment ^a^	-	-	-	5 (20)	2 (40)	0.56	-	-	-
Antibiotic classes:									
Nonantipseudomonas β-lactams only	7 (4.3)	0	0.66	0	0	-	7 (5.1)	0	1
Antipseudomonas β-lactams	51 (31.5)	7 (53.8)	0.13	17 (68)	2 (40)	0.33	34 (24.8)	5 (62.5)	0.03 *
Macrolide	74 (45.7)	5 (38.5)	0.61	13 (52)	2 (40)	1	61 (44.5)	3 (37.5)	0.73
Quinolone only	17 (10.5)	2 (15.4)	0.64	3 (12)	1 (20)	1	14 (10.2)	1 (12.5)	1
Atypical pathogen coverage	98 (60.5)	8 (61.5)	0.94	17 (68)	3 (60)	1	81 (59.1)	5 (62.5)	1
Guideline concordant Therapy ^b^	75 (46.3)	6 (46.1)	0.99	14 (56)	2 (40)	0.64	61 (44.5)	4 (50)	1
Duration of initial antibiotics treatment, median (IQR), d	6.5 (4–7)	6 (4.5–7)	0.56	7 (5.5–7.5)	6 (4.5–9.5)	0.32	6 (3–7)	7 (3.7–7)	0.34
Duration of initial antibiotics treatment < 5 days	55 (33.9)	3 (23.1)	0.55	3 (12)	1 (20)	1	52 (37.9)	2 (25)	0.71

Data are presented as mean ± SD or n (%), unless otherwise stated. ^a^ Pathogens detected were not susceptible to the antibiotics administered within 24 h of presentation. ^b^ The 2019 ATS/IDSA guideline on the management of community-acquired pneumonia in adults. * Statistically significant difference.

## Data Availability

Not applicable.

## Supplementary Materials

The following supporting information can be downloaded at: https://www.mdpi.com/article/10.3390/tropicalmed8030148/s1, Table S1: American Thoracic Society/Infectious Diseases Society of America criteria for defining severe community-acquired pneumonia; Table S2: American Thoracic Society/Infectious Diseases Society of America Guideline Recommendations for empirical therapy for community-acquired pneumonia; Table S3: Initial antibiotics treatment and hospital-free days within 30 days after hospital admission of all patients hospitalized with respiratory syncytial virus-associated acute respiratory illness.

## Abbreviations

ARI	acute respiratory illness
CAP	community-acquired pneumonia
CI	confidence interval
eGFR	estimated glomerular filtration rate
FiO_2_	fraction of inspired oxygen
HR	hazard ratio
aHR	adjusted hazard ratio
HAP	hospital-acquired pneumonia
ATS/IDSA	American Thoracic Society/Infectious Diseases Society of America
IQR	interquartile range
OR	odds ratio
RSV	respiratory syncytial virus
RT-PCR	reverse transcription polymerase chain reaction
SD	standard deviation
VAP	ventilator-associated pneumonia
