# Supplementary material for: Bacterial Coinfection and Superinfection in Respiratory Syncytial Virus-Associated Acute Respiratory Illness: Prevalence, Pathogens, Initial Antibiotic-Prescribing Patterns and Outcomes
Source: Trop Med Infect Dis. 2023 Feb 27;8(3):148. doi: 10.3390/tropicalmed8030148 (PMC10057067; doi:10.3390/tropicalmed8030148)
Supplement: Supplementary file 1 [file tropicalmed-08-00148-s001.zip › tropicalmed-2158026-supplementary.pdf]

Tropical Medicine and Infectious Disease

Supplementary Materials

Bacterial Coinfection and Superinfection in Respiratory Syncytial Virus-Associated Acute Respiratory Illness: Prevalence, Pathogens, Initial Antibiotic-Prescribing Patterns and Outcomes

Phunsup Wongsurakiat [1,*], Siwadol Sunhapanit [2] and Nisa Muangman [3]

[1] *Division of Respiratory Diseases* and Tuberculosis, *Department of Medicine,* Faculty of Medicine Siriraj Hospital, *Mahidol University,* 2 Wanglang Road, *Bangkoknoi, Bangkok 10700, Thailand*
[2] Division of Pulmonary Medicine and Pulmonary Critical Care, Faculty of Medicine, Vajira Hospital, Navamindradhiraj University, Bangkok 10300, Thailand; siwadol2530@gmail.com
[3] Diagnostic Division, Department of Radiology, Faculty of Medicine Siriraj Hospital, *Mahidol University,* 2 Wanglang Road, *Bangkoknoi, Bangkok 10700, Thailand; mailtonisa@gmail.com*
* Correspondence: phunsup.won@mahidol.ac.th

Table S1. American Thoracic Society/Infectious Diseases Society of America criteria for defining severe community-acquired pneumonia [1].

Major criteria	Minor criteria
• Respiratory failure requiring invasive mechanical ventilation<br>• Septic shock with need for vasopressors	• Confusion/disorientation<br>• Hypotension requiring aggressive fluid resuscitation<br>• PaO_2/FiO_2 ratio $\leq$ 250 mmHg<br>• Respiratory rate $\geq$ 30 breaths/min or need for non-invasive ventilation<br>• Multilobar infiltrates<br>• Hypothermia (core temperature < 36°C)<br>• Blood urea nitrogen level $\geq$ 20 mg/dL)<br>• Leukopenia (white blood cell count < 4,000 cells/mm^3)<br>• Thrombocytopenia (platelet count < 100,000 cells/mm^3)
**Definition includes $\geq$ 1 major criterion or $\geq$ 3 minor criteria**	

Table S2. American Thoracic Society/Infectious Diseases Society of America Guideline Recommendations for empirical therapy for community-acquired pneumonia [1].

**Inpatients, non-ICU treatment**
A nonantipseudomonal β-lactam[a] plus a macrolide[b]
A respiratory fluoroquinolone[c]

**Inpatients, ICU treatment**
A nonantipseudomonal β-lactam[a] plus either azithromycin or a respiratory fluoroquinolone[c]
**If *Pseudomonas* is a concern**
An antipneumococcal, antipseudomonal β -lactam[d] plus either ciprofloxacin or levofloxacin
or
The above β-lactam plus an aminoglycoside[e] and azithromycin
or
The above β-lactam plus an aminoglycoside[e] and a respiratory fluoroquinolone[c]

ICU: intensive care unit.

aCefotaxime, ceftriaxone, ampicillin/sulbactam, or ertapenem

bAzithromycin, clarithromycin, or erythromycin

c Levofloxacin, or moxifloxacin

d Piperacillin-tazobactam, cefepime, imipenem, or meropenem

e According to Thai guidelines for the management of adults with community-acquired pneumonia, adding an aminoglycoside is optional. An aminoglycoside may be added to the initial antibiotic regimens only if multi-drug resistant Pseudomonas infection is suspected.

Table S3. Initial antibiotics treatment and hospital-free days within 30 days after hospital admission of all patients hospitalized with respiratory syncytial virus-associated acute respiratory illness.

	All Patients n = 175		Bacterial coinfection n = 30		No bactarial coinfection n = 145	
	Hospital free days[a]	*p* value	Hospital free days[a]	*p* value	Hospital free days[a]	*p* value
Initial antibiotic treatment:		0.06		-		0.19
Yes	20 (10.7-23)		16 (3-20.2)		21 (13-24)	

	(n = 150)		(n = 30)		(n = 120)	
No	22 (18-25)		-		22 (18-25)	
	(n = 25)		(n = 0)		(n = 25)	
Inadequate initial antibiotic	-	-		.8	-	-
treatment[b]:			17 (4 to 20)			
Yes			(n = 23)			
			9 (0 to 23)			
No			(n = 7)			

Antibiotic classes:						
Nonantipseudomonas β-lactams		0.85		-		0.94
only:	20 (14-27)		-		20 (14-27)	
Yes	(n = 7)		(n = 0)		(n = 7)	
	20 (13-23)		16 (3-20.2)		21 (14.7-24)	
No	(n = 168)		(n = 30)		(n = 138)	
Antipseudomonas β-lactams:		0.009*		0.5		0.11
Yes	18.5 (5-22.2)		15 (5-19)		20 (6-23)	
	(n = 58)		(n = 19)		(n = 39)	
No	21 (15.5-24)		19 (0-23)		21 (16-24)	
	(n = 117)		(n = 11)		(n = 106)	
Macrolide:		0.42		0.57		0.55
Yes	20 (9-24)		9 (0-21)		20 (12.2-24)	

	(n = 79)		(n = 15)		(n = 64)	
No	20.5 (16-23)		17 (9-20)		21 (17-24)	
	(n = 96)		(n = 15)		(n = 81)	
Quinolone only:		0.63		0.83		0.63
Yes	20 (13-22)		16.5 (3.2-21.5)		20 (13-22)	
	(n = 19)		(n = 4)		(n = 15)	
No	20 (13-24)		16 (3-20.2)		21 (14.7-24)	
	(n = 156)		(n = 26)		(n = 130)	
Atypical pathogen coverage:		0.19		0.58		0.29
Yes	20 (9.7-23.2)		12.5 (1-20.7)		20 (12.7-24)	
	(n = 105)		(n = 20)		(n = 86)	
No	21 (17-23.5)		18 (6.7-20.7)		21 (18-24)	
	(n = 69)		(n = 10)		(n = 59)	
Guideline concordant therapy[c]:		0.37		0.36		0.73
Yes	20 (7-24)		10.5 (1-19.7)		21 (10.5-24)	
	(n = 81)		(n = 16)		(n = 65)	
No	20 (16-23)		18 (6.7-22.2)		21 (17-23)	
	(n = 94)		(n = 14)		(n = 80)	
Duration of initial antibiotics <5	23.5 (19-25.2)	<0.001*	21.5 (4.7-24.7)	0.22	23.5 (19-26)	<0.001*
days	(n = 58)		(n = 4)		(n = 54)	
	19 (8-22)		14 (3-20)		20 (12-22)	

Duration of initial antibiotics ≥5 days	(n = 117)	(n = 26)	(n = 91)

Data are presented as mean ± SD or n (%), unless otherwise stated. [a]Number of days from admission to day 30 that the patient was not admitted to the hospital. [b]Pathogens detected were not susceptible to the antibiotics administered within 24 h of presentation. [c]The 2019 ATS/IDSA guidelines on the management of community-acquired pneumonia in adults. *Statistically significant difference.

References

1. Metlay, J.P.; Waterer, G.W.; Long, A.C.; Anzueto, A.; Brozek, J.; Crothers, K.; et al. Diagnosis and Treatment of Adults with Community-acquired Pneumonia. An Official Clinical Practice Guideline of the American Thoracic Society and Infectious Diseases Society of America. *Am. J. Respir. Crit. Care Med*. **2019**, 200(7), e45-e67.
